# A Patient with Acute Abdominal Pain Caused by an Unnoticed Swallowed Toothpick Misdiagnosed as Acute Appendicitis

**DOI:** 10.34172/aim.2022.44

**Published:** 2022-04-01

**Authors:** Yong Yao, Gaowu Yan, Lei Feng

**Affiliations:** ^1^The Division of Gastroenterology and Hepatology, Suining Central Hospital, Suining City, Sichuan Province, China; ^2^The Department of Radiology, Suining Central Hospital, Suining City, Sichuan Province, China

**Keywords:** Acute abdominal pain, Acute appendicitis, Computed tomography, Differential diagnosis, Foreign body, Toothpick

## Abstract

The differential diagnosis of acute abdominal pain is a challenging task for medical doctors working in the department of gastroenterology. It is clear that acute abdominal pain may be associated with a number of pathologic conditions. We report an unusual case of an unnoticed swallowed wooden toothpick stuck in the ileocecal area of a young man with right lower abdominal pain who was misdiagnosed as acute appendicitis. However, an abdominal computed tomography scan showed an elongated foreign body stuck in the ileocecal area. The elongated foreign body was identified as a wooden toothpick, which was then grasped with a foreign body forceps and successfully removed through colonoscopy. The patient’s abdominal pain was significantly relieved within 2 days following treatment. On the basis of the case report, we suggest the importance of abdominal computed tomography scans for the differential diagnosis of acute abdominal pain and highlight the need for extra vigilance in excluding the diagnosis of foreign bodies in the gastrointestinal tract of patients with acute abdominal pain.

## Introduction

 Acute abdominal pain is a common symptom among patients who present to the department of gastroenterology, which can be caused by various diseases, including acute appendicitis, bowel obstruction, nonspecific abdominal pain, acute diverticulitis and gastro-intestinal diseases.^1^ The different causes for acute abdominal pain can entail different prognoses, ranging from self-limiting to life-threatening processes among patients with acute abdominal pain.^2^ Acute appendicitis, a life-threatening disease, is manifested by acute right lower abdominal pain, fever, nausea, vomiting and diarrhea and often accompanied by the development of severe complications.^3^ Most patients with acute appendicitis require timely diagnosis and surgical treatment for better outcomes. However, the diagnosis of acute appendicitis is based on history and typical clinical symptoms and some diseases presenting as acute right lower abdominal pain may be easily mistaken for acute appendicitis.^4^ Here, we report a case of acute right lower abdominal pain due to an unnoticed swallowed toothpick, which was misdiagnosed as acute appendicitis and successfully treated by endoscopic removal of the toothpick.

## Case Report

**Figure 1:**
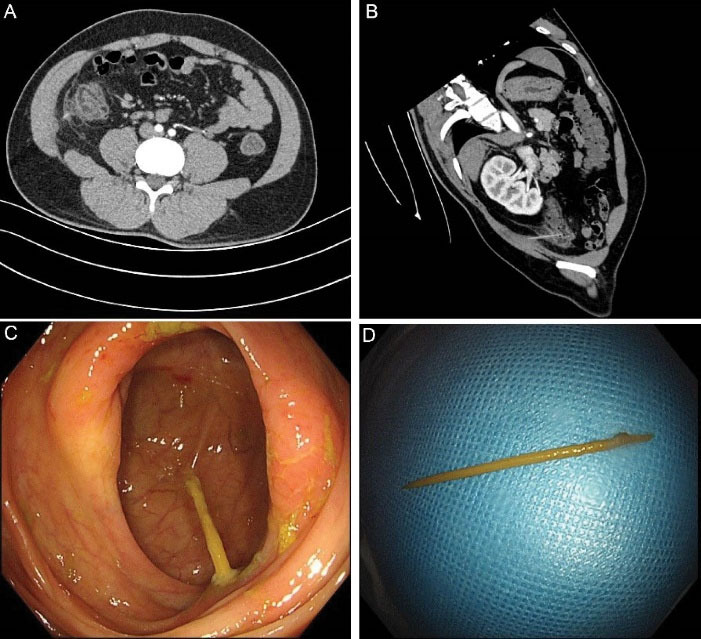
A Toothpick Stuck in the Ileocecal Region of a Patient with Right Lower Quadrant Abdominal Pain. **(A)** CT scan of the abdomen reveals a foreign body. **(B) **CT scan sagittal reconstruction image shows a high-density needle-shaped structure in the ileocecal area.** (C) **Endoscopic view of the toothpick stuck in the ileocecal area. **(D) **Photograph of the removed wooden toothpick.

 A 27-year-old young man was admitted to our hospital as a patient presenting with a 3-day history of right lower abdominal pain and diarrhea. He had a 10-pack-year history of cigarette smoking and a history of habitual heavy alcohol intake of more than 70 g/d. The rest of the patient’s history, family history, developmental history, social history, and review of systems was unremarkable. Physical examination showed that he had a normal body temperature and localized right lower abdominal tenderness with mild rebound tenderness, but no guarding. Blood counts revealed a mild leukocytosis 11.5 × 10^9^/L, with predominant neutrophils (76.2%) with normal platelet count and hemoglobin. Plasma high-sensitivity C-reactive protein was increased at 2.99 mg/dL. Computed tomography (CT) of the abdomen without contrast media revealed swelling of the ileocecal mucosa. Other laboratory tests and conventional radiological examinations were normal. Therefore, a primary diagnosis of acute appendicitis was made based on his history and clinical examination findings. However, his infections were unapparent or mild, and thus, he underwent a contrast-enhanced CT scan of the abdomen. Interestingly, the CT scan of the abdomen revealed the presence of an elongated foreign body in the ileocecal area (Figures 1A and 1B). Accordingly, the patient was examined by colonoscopy and the results of colonoscopy showed a wooden toothpick stuck in the ileocecal area (Figure 1C). The wooden toothpick was then grasped with a foreign body forceps and successfully removed through colonoscopy (Figure 1D). The young man was unaware of ever having swallowed a wooden toothpick, but remembered that he had pitched up food with a toothpick in a state of drunkenness 3 days ago. The patient’s abdominal pain was significantly relieved within 2 days following treatment.

## Discussion

 Here, we report a rare case of foreign body stuck in the ileocecal area, which was misdiagnosed as acute appendicitis. Various laboratory tests and radiological examinations are available to assist the diagnosis of acute appendicitis, but none of them are specific for the documentation of acute appendicitis.^5,6^ Therefore, the differential diagnosis of acute appendicitis is challenging, especially in young women for whom there is a larger range of differential diagnoses than for men.^7^ Gottschalk et al reported a case of an incarcerated uterus in a patient presenting at 24 weeks’ gestation with severe bilateral flank and lower abdominal pain who was initially misdiagnosed as acute appendicitis.^8^ For this reason, acute appendicitis should be diagnosed by ruling out other causes of right lower abdominal pain. CT is an important tool in the diagnosis and differential diagnosis of acute appendicitis.^9-11^ Sinnott andHowlett reported a case of a 38-year-old man with acute appendicitis who presented with atypical clinical symptoms and underwent a CT scan, which showed a dilated, thick walled appendix, confirming the diagnosis of acute appendicitis.^12^ Previous studies for patients with acute appendicitis have shown that CT has a high level of diagnostic accuracy that is unaffected by the percentage of female participants, adopted scanning protocol or patient presentation. CT has proven to be more accurate in the diagnosis of acute appendicitis than ultrasonography and has specificity and sensitivity rates between 83–100% and 76–100%.^13^ Abdominal CT scan in patients with suspected acute appendicitis can significantly reduce the rate of negative appendectomy, which is as high as 15%.^14^ Accordingly, abdominal CT should be performed as a routine preoperative procedure in all patients with suspected acute appendicitis.

 In our patient, abdominal CT was performed to reveal that right lower abdominal pain in this patient was likely the result of an elongated foreign body stuck in the ileocecal area and colonoscopy confirmed that the foreign body was a wooden toothpick. So far, a number of case reports have presented the cases of foreign bodies in the gastrointestinal tract of patients with various clinical manifestations. Venkatesan et al reported a case of pyogenic hepatic abscess secondary to gastric perforation caused by an ingested fish bone.^15^ Unexpectedly, a mobile phone was found in the stomach of a 39-year-old man.^16^ To the best of our knowledge, ours is the first report to present a case of an unnoticed swallowed wooden toothpick stuck in the ileocecal area of a young man with right lower abdominal pain who was misdiagnosed as acute appendicitis. Because the small intestine of an adult is about 6.4 meters long, a swallowed toothpick is often difficult to reach the ileocecal area through the small intestine. A previously published study reported a patient who suffered from a toothpick stuck in small intestine,^17^ but in our case, the toothpick reached the ileocecal area.

 In conclusion,this case emphasizes the importance of an abdominal CT scan in the clinical treatment of abdominal pain. In addition, the diagnosis of foreign bodies in the gastrointestinal tract should be considered in the differential diagnosis of abdominal pain.
